# Factors Influencing Sustained Utilization of Child Welfare Services among Children Aged 18 to 59 Months in a Low-Income Rural Community, Ghana

**DOI:** 10.1155/2021/1803946

**Published:** 2021-03-23

**Authors:** Kennedy Diema Konlan, Roberta Mensima Amoah, Abdul Razak Doat, Juliana Asibi Abdulai

**Affiliations:** ^1^University of Health and Allied Sciences, School of Nursing and Midwifery, Department of Public Health Nursing, Ho, Volta Region, Ghana; ^2^University for Development Studies, School of Allied Health Sciences, Tamale, Ghana; ^3^Nursing and Midwifery Training College, Tamale, Northern Region, Ghana; ^4^Department of Surgery, Tamale Teaching Hospital, Tamale, Northern Region, Ghana

## Abstract

**Background:**

Despite substantial progress in reducing child mortality, concerted efforts remain necessary to avoid preventable deaths in children under-5 years and to accelerate progress in improving child survival. The patronage of child welfare services is paramount to the attainment of these goals. This study identified the factors that influence the patronage of child welfare services in a rural community in the Ho West District of the Volta region. *Methodology*. This quantitative descriptive cross-sectional design employed a systematic sampling method to select 310 caregivers of children aged 18 to 59 months in the Abutia Kloe subdistrict using a pretested questionnaire. The data were entered into a Microsoft excel spreadsheet and cleaned and exported to Statistical Package for Social Sciences (SPSS 22) for analysis.

**Results:**

The results showed that children (44.2%) had defaulted at a point during the continued growth monitoring process. The reasons for the default included completed major immunization (72.3%), started school (57.4%), and poor staff attitude (3.2%). Mothers have an idea about the purpose of the growth chart (68.0%) as the mothers (86.5%) are able to access a child welfare clinic in less than thirty minutes' walk from their homes. The cross tabulation on level of education and regular CWC attendance showed a strong association (*r*^2^ = 8.071, *p* ≤ 0.03). Cross tabulation on marital status and CWC attendance showed a positive significant association (*r*^2^ = 17.307, df = 2, *p* ≤ 0.001). Married caregivers (85.2%) as compared with unmarried ones (60.5%) are more likely to seek child welfare services for their child.

**Conclusion:**

Healthcare providers should intensify education on the need to continue growth monitoring up to 59 months even after the completion of major immunization. This goal can be attained if growth monitoring is incorporated into school health activities while policy implementers ensure the full execution.

## 1. Introduction

The health and wellbeing of children under 59 months have been an issue of concern for decades, and it is estimated that more than six million children still die before their fifth birthday each year [1] and one in every nine Ghanaian children dies before reaching age five [2]. Several interventions have been implemented as part of the Sustainable Development Goal (SDG) three to improve the health of individuals of all ages including children under-five years of age and to reduce under-5 mortalities [3]. Despite substantial progress in reducing child mortality, concerted efforts remain necessary to avoid preventable deaths in children aged under-5 in the coming years and to accelerate progress in improving child survival. Some of the interventions currently in place to reduce under-5 child mortality include the expanded programme for immunization including micronutrient supplementation and the Integrated Management of Childhood and Neonatal Illness (IMCI) [4, 5]. An increasing proportion of child deaths is noted in sub-Saharan Africa and southern Asia, and four out of every five deaths of children under-5 years occur in these regions [1]. This inequality in the health of children under-5 years has been attributed to weak healthcare systems in most of the African countries [6]. This has energised most countries to implement primary healthcare interventions that encourage service provision in homes of beneficiaries.

In Ghana, an essential part of the primary healthcare (PHC) system is the establishment of child welfare clinics (CWCs) all over the country [7]—under the community health planning and services (CHPSs) initiative. Children under-5 years are assessed at CWC for various child welfare and health services. Among the child health activities at the CWC are growth monitoring, immunization against childhood killer diseases, vitamin A supplementation, treatment of minor ailments, and referral of complicated illnesses, health talks, and counselling of caregivers [4]. The clinic treating children under-5 years combines preventive, treatment, health surveillance, and education into a system of comprehensive healthcare [4, 8]. Caregivers of children are encouraged to attend CWC after delivery to benefit from PHC services including child immunizations and growth monitoring and promotion—commonly termed “weighing.” However, about 70.0% of districts and regions have challenges with effective coverage of childhood immunization, and a majority of children under-5 years do not fully receive components of the child welfare services (CWSs) including immunization and growth monitoring [9]. A study conducted in Techiman to evaluate the immunization coverage among children older than 12 months revealed that around 80.0% of children between 12 and 23 months were immunized; however, it was revealed that most of these children were partially immunized, and some were not immunized at all [10]. Patronage of CWC services including immunization of children and regular growth monitoring of children in Ghana has remained low [11]. Statistics from the Ghana health service shows a consistent decline in the CWC attendance, and this decline has been attributed to knowledge gaps among caregivers and a lack of defaulter tracing systems in most CWCs [12]. While this remains a national reason for the decline or limited utilization of CWC services, this study assessed those specific factors in low-income rural communities that influence the utilization of CWC services.

## 2. Methodology

### 2.1. Study Design

A descriptive cross-sectional design was used as data were collected from participants only once and no further follow-up was done.

### 2.2. Population, Sample, and Sampling Technique

The study was conducted in Ho West District of Ghana which is one of the twenty-five districts of the then Volta Region with a land area of 1,002.79 square kilometres before the Oti region was carved out. The total population of the district was 94,600, comprising 45,361 males with a total fertility rate of 3.6 [12]. The crude birth rate of the district was 24.2 per 1000 population with a crude death rate of 12.5 per 1000. The indigenes form 70.0% of the population. The district has six health administrative subdistricts. Abutia (one of the subdistricts) community is largely a sparsely populated community with many of its inhabitants being farmers and petty traders. The majority of children in this community are in school, while those not in school are engaged in helping their caretakers/parents with menial jobs in the farm or market.

The research population comprised caregivers of children aged 18 to 59 months residing in Abutia and its environs. The estimated population of children 18 to 59 months in the community was estimated by the district health directorate to be 1,348 [7].

The sample size was determined using the following equation: *n* = *N*/(1 + *N* (*e*^2^) [13], where *n* = sample size, *N* = study population size (1348), and *e* = sampling error (at 95.0% confidence level). The systematic sampling method was used to select 310 caregivers of children less than 60 months in the catchment area of the Abutia Kloe subdistrict.

### 2.3. Data Collection and Analysis

A sample fraction of 4 was computed, and based on that, respondents were recruited. In the study, community researchers in each community were dispatched in all directions to ensure total coverage of the communities. Study participants were recruited from homes, farms, and sometimes in market stalls. It took an average of 10 minutes to complete each research questionnaire. Items in the questionnaire were both closed-ended and open-ended. Information on sociodemographic indices, knowledge on the purpose of growth chart and child welfare services, and availability and coverage of child welfare services was collected.

The data were entered into a Microsoft Excel Spreadsheet and cleaned and subsequently exported to the statistical package for social sciences (SPSS -version 22.0) for analysis. Data were analysed into simple descriptive statistics and tests of associations. A *p* value < 0.05 was considered as statistically significant at a 95.0% confidence interval.

### 2.4. Ethics Clearance

Ethical approval was obtained from the Research and Ethics Committee of the institute of health research of the University of Health and Allied Health Sciences (UHAS.REC/A.5[24]17–18). Upon approval from the institutional Ethics Review Board, permission was sought from Abutia Kloe community leaders and health service administrators to conduct the study. Informed consent was also sought from mothers before they voluntarily participated in the study. The respondents were informed of their right to participate or withdraw from the study without any repercussions.

## 3. Results

The study respondents for this study were 310 mothers, as 58.4% were aged between 21 and 30 years and 36.1% between 31 and 40 years, as those aged less than 20 years and more than 40 years were 2.6% and 2.9%, respectively. Also, 87.1% were unemployed, 10.3% employed, and 2.6% students. Respondents have had formal education (82.0%) at least up to the primary school level as 17.6% never had formal education (Table 1).

Describing the specific age, the mothers think the child should stop CWS, and the response showed less than 2 years (8.0%), 2 years (40.0%), 3 years (32.0%), 4 years (17.0%), and above 4 years (3.0%). Caretakers of children indicated that their children have not received all the CWC services corresponding to their ages (44.2%), as 2.6% could not tell whether their babies had received all the appropriate services for their age, while 53.2% received all CWC services corresponding to their ages. The main reasons for default were completion of the major immunization (72.3%), children starting school (25.0%), and poor attitude of nurses (3.2%). The main reasons for default of CWS were completion of the major immunization (72.3%), children starting school (25.0%), and poor attitude of nurses (3.2%). The most important factor that can determine the stoppage of CWS in children, the respondents indicated that when the mother gets pregnant (32.0%), any time the mother feels like (42.0%), and at the advice of the health worker (26.0%).

Caretakers of children between 18 and 59 months had fair knowledge on the available services at the child welfare clinic. These services at the CWC included weighing of babies (23.3%), vaccination (22.6%), vitamin A supplementation (20.6%), health education (15.6%), and counselling (17.6%). A good proportion of the mothers (27.1%) did not know what declining weight record of their children meant. The meaning of weight decline by caregivers however was stated as the child has lost weight (28.7%), the child is sick (23.0%), or the child has not been eating well (21.2%). The meaning of weight decline by caregivers however was stated as the child has lost weight (28.7%), the child is sick (23.0%), or the child not eating well (21.2%). The mothers (63%) indicated that the children will need to continue with monthly weighing even as they have completed the recommended immunisation schedule. Describing the most important way to improve CWS in the area, mothers indicated that CHNs should visit their homes to provide the service (34.0%), the service should be provided bimonthly (15.0%), mothers who compile with the service should be awarded (21.0%), CWS should be incorporated as a school health activity (22.0%), and mothers should be empowered to understand and interpret the weight record chart (8.0%).

In Table 2, the cross tabulation on level of education and regular CWC attendance showed a strong association (*r*^2^ = 8.071, df = 3, *p* ≤ 0.03). Responses showed that none educated (80.0%) compared to educated (93.4%) mothers were more likely to send their child for child welfare care services. Cross tabulation on marital status and CWC attendance showed a positive significant association (*r*^2^ = 17.307, df = 2, *p* ≤ 0.001). The results showed that married caregivers (85.2%) as compared with unmarried ones (60.5%) are more likely to seek child welfare services. Cross tabulation on respondents' number of children and regular attendance of CWC showed a weak association (*p* ≤ 0.244). The relationship between the number of children and regular attendance of CWC is presented in Table 2.

## 4. Discussion

The level of child welfare clinic coverage among children under-five years in a rural community in Ghana was assessed, and it was shown that 44.2% of the respondents had not received all the CWC services appropriate for their ages. The World Health Organization (WHO) guideline makes it clear that every child should continue and complete the growth monitoring at the age of 59 months to get the maximum benefit to ensure the child's survival [5]. Abutia Kloe located in the Ho West District is one of the 70.0% districts in Ghana where the majority of children under-5 years do not fully receive components of the CWS including immunization and growth monitoring [9]. The default rate for CWC services was shown to be 72.3% which is higher than the recommended default rate of CWC services expected by the Ghana health service [12]. The reasons espoused for CWC default included completion of immunization schedule (10.2%). Other studies in sub-Saharan Africa showed that mothers consider only immunization services to be the sole reason for the provision of CWS and, upon its completion, quits CWC. Some mothers (47.0%) indicated that monthly weighing is not important as they could not identify any immediate tangible benefits associated with the service. It is prudent that mothers have continuous education on the importance of growth monitoring as a crucial component of CWC.

Another reason for CWC default was because children start school earlier (25.0%) than the expected period for completion of the CWC services. CWC services are provided in primary healthcare institutions that largely provide services during working/schooling hours. School health services were introduced by the Ghana health services to provide these essential services to school children as a school nurse was posted to some schools, but this service has not largely been patronised in the Ghanaian context and still leaves much to be desired [14]. The importance of school healthcare services is further emphasized as the nature of work of caregivers, and having limited time (31.3%) was cited as the reason for default for CWC services. In Ghana, parents having limited time were the very reason that was only second to completion of immunization as a reason for default of CWC [14]. The mothers' preoccupation could be attributed to the fact that most mothers worked in their farms as this assertion is supported as nurses mentioned that mothers fail to turn up because they are always busy in their farms [14].

Studies in Southern Ghana and Ethiopia made a similar finding where infants who attended primary school could not complete all aspects of CWS [14–17]. In line with this finding, integrating CWS in early childhood school will support in reducing the incidence of defaulting. When mothers lack information about CWS schedules, it affects coverage. Some mothers in the current study did not know that CWS lasts for 5 years and might therefore stop when the child is healthy and is not due for any of the specialized CWS. Studies conducted in Ghana, Central Pakistan, Ethiopia, and India confirm that patronage is affected when mothers lack information [14–17]. The lack of information about vaccination schedules and service arrangements, including unusual circumstances such as what to do after missed appointments, resulted in default [18]. Few mothers (3.2%) cited poor staff attitude as a reason for defaulting CWC. It is imperative that healthcare providers ensure a sustained nonjudgemental attitude towards clients and babies especially during the provision of preventive services like CWS. In Kenya, mothers especially those with malnourished children complained of such bad attitude toward them [19], and in Ethiopia, mothers reported fear of mistreatment and lack of cooperation from service providers [18]. In the Ga West Municipality of Ghana, an unprofessional attitude of staff hindered service utilization [20].

Mothers' knowledge on services provided for children at the CWC to include weighing (23.3%), vaccination (22.6%), vitamin A supplementation (20.6%), health education (15.6%), and counselling (17.6%). The majority of mothers have had apt knowledge on the type of services at the CWC. In Ghana, a significant portion (84.0%) of respondents was able to mention at least three services provided to children at CWC [14]. With regards to the purpose of growth chart and its interpretation, 32.0% of the mothers did not know the purpose of growth chart as 47.1% could not properly interpret the meaning of the growth chart. The growth chart is designed graphically for mothers to be able to interpret the results; thus, mothers could serve as comonitors of the progress attained by their children. This is significant because mothers who know the health benefits of growth monitoring and understand the information displayed on the child health card are more likely to practice continued growth monitoring [21]. It is therefore necessary that health education on growth charts is to be intensified to increase participation to achieve the Ghana Health Service expectation that every child aged 59 months completes the CWC in order to get the maximum benefit to ensure the child's survival [22].

Distance plays a significant role in the utilization of healthcare services. The majority (86.5%) of the mothers reached CWS centres in less than 30 minutes as 20.6% of the respondents cited that distance hindered full participation in CWS. The community-based health planning and services (CHPS) has helped provide primary healthcare services to hard-to-reach communities and reduced significantly the distance covered in order to access primary healthcare services. Mothers who walk over a long distance are more likely to default from services. In Ghana and Togo, children whose parents had to walk half an hour to one hour to reach a healthcare centre were 57.0% more likely to have an incomplete coverage than those whose parents had to walk less than half an hour [14, 23]. In Kenya, for example, the availability of health facilities within 5 km return journey influenced the practice of continued growth monitoring [21]. Most of the mothers (83.9%) who sort CWC services got to the health facility walking. Drop out increased in families with low income, whereas the likelihood of incomplete growth monitoring decreased with increase in household's income [23–25]. Healthcare providers must be stationed closer to the target communities to reduce the likelihood of spending additional money on transport in seeking services within these particular communities.

The higher the educational level of the mother, the more likely it is for the mother to participate in continuous sustained CWS (*r*^2^ = 8.071, *p* ≤ 0.03). Educated mothers are more likely to read and comprehend the child health record book and, as a result, will participate positively to the attainment of good health outcomes. The educated mothers were less likely to default as compared to mothers with no or only basic education in Ghana, Togo, Ethiopia, Nigeria, Senegal, Bangladesh, and India [14, 17, 23, 24, 26]. Female literacy is vital in increasing CWS patronage and to increase participation among mothers attending CWS. Mothers were more likely not to default CWS if they were married, indicating that the involvement of the father/husband might play a decisive role in increasing CWS coverage (*r*^2^ = 17.307, df = 2, *p* ≤ 0.001). In Ghana and Kenya where marriage had the potential of enhancing attendance of continued CWS, husbands when provided with health education appear to be willing to assist and encourage their wives to take their children for CWS at the health facilities [10, 18, 21]. In light of this finding, it is necessary to investigate the educational level of husbands when assessing regular participation in CWS. This is prudent because Asirifi [27] reported that low levels of formal education influence healthcare delivery in Ghana.

## 5. Conclusion

The majority of mothers of the Abutia Kloe community do not understand the information displayed on the growth monitoring chart of children accessing CWS. Healthcare implementers should focus on educating mothers and caregivers on how to read and interpret the growth monitoring charts using the colour codes displayed in the growth monitoring chart book. Policymakers should look at intensifying education on the need to continue growth monitoring up to 59 months because mothers are likely to default on completion of immunization schedule. Ways to integrate growth monitoring with school's healthcare services should be looked at in order to ensure continued growth monitoring even though students are in school.

## Figures and Tables

**Table 1:** Demographic characteristics and caregivers' perception towards CWS.

Variables	Parameter	Frequency	Percent
Age distribution	<20	8	2.6
	21–30	181	58.4
	31–40	112	36.1
	>40	9	2.9

Distribution of education status	None	45	14.5
	Basic	181	58.4
	Secondary	44	14.2
	Tertiary	40	12.9

Employment status	Employed	32	10.3
	Unemployed	270	87.1
	Student	8	2.6

Perceptions of services provided at CWC	Weighing	302	23.32
	Vaccination	294	22.63
	Vitamin A supplementation	267	20.55
	Health education	203	15.63
	Counselling	229	17.63

Meaning of declining weight record	Child is not growing well/has lost weight	145	28.71
	Child may be sick/has been sick	116	22.97
	Child is not eating well	107	21.19
	Do not know	137	27.13

**Table 2:** Cross tabulation of demographic characteristics and CWC attendance.

Variables	Regular CWC attendance			Pearson chi-square correlation			
		Yes	No		Value	Df	*pvalue*

Level of education and CWC attendance	None	36	9	*R* ^2^	8.071	3	0.045
	Basic	146	35	Likelihood ratio	13.658	3	0.003
	Secondary	34	10	Linear-by-linear assoc.	3.508	1	0.061
	Tertiary	32	0	*N* of valid cases	302		

Number of children and CWC attendance	Married	213	37	*R* ^2^	17.307	2	0.001
	Not married	26	17	Likelihood ratio	16.302	2	0.001
	Separated	9	0	Linear-by-linear assoc.	1.266	1	0.261

Marital status and CWC attendance	Married	213	37	*R* ^2^	17.307	2	0.001
	Not married	26	17	Likelihood ratio	16.302	2	0.001
	Separated	9	0	Linear-by-linear association	1.266	1	0.261
	Total	248	54	*N* of valid cases	302		

## Data Availability

All data generated or analysed during this study are included in this published article, and no data set is deposited in any data repository.
